# The development of pGALSplus: evaluating feasibility and acceptability of an assessment to facilitate the identification and triage of children with musculoskeletal presentations

**DOI:** 10.1093/rap/rkae089

**Published:** 2024-08-01

**Authors:** Vicky Mercer, Nicola Smith, Michela Guglieri, Simon A Jones, Jeremy R Parr, Helen E Foster, Sharmila Jandial

**Affiliations:** Translational and Clinical Research Institute, Newcastle University, Newcastle upon Tyne, UK; Children’s Physiotherapy, South Tyneside and Sunderland NHS Foundation Trust, South Shields, UK; Department of Sport, Exercise and Rehabilitation, Faculty of Health and Life Sciences, Northumbria University, Newcastle upon Tyne, UK; Translational and Clinical Research Institute, Newcastle University, Newcastle upon Tyne, UK; John Walton Muscular Dystrophy Research Centre, Newcastle University and Newcastle Hospitals NHS Foundation Trust, Newcastle upon Tyne, UK; Manchester Centre for Genomic Medicine, Saint Mary’s Hospital, Manchester, UK; Population Health Sciences Institute, Newcastle University, Newcastle upon Tyne, UK; Great North Children’s Hospital, Newcastle upon Tyne Hospitals NHS Foundation Trust, Newcastle upon Tyne, UK; Emerita Professor Paediatric Rheumatology, Population Health Sciences Institute, Newcastle University, Newcastle upon Tyne, UK; School of Medicine, Newcastle University, Newcastle upon Tyne, UK; Paediatric Rheumatology, Great North Children’s Hospital, Newcastle upon Tyne, UK

**Keywords:** pGALS, musculoskeletal, children, assessment, allied health, rheumatology, development, muscle disease

## Abstract

**Objectives:**

Healthcare professionals (HCPs) need to identify potentially serious musculoskeletal (MSK) presentations in children and refer them to specialists appropriately. Our aim was to develop ‘pGALSplus’ (paediatric gait, arms, legs and spine plus) to support clinical assessment, aid decision-making and assess feasibility and acceptability in exemplar MSK pathologies.

**Methods:**

We used a three-phase mixed methods approach: phase 1, preliminary stakeholder engagement and scoping review to propose pGALSplus; phase 2, iterative development of pGALSplus involving an expert working group; and phase 3, testing the feasibility of pGALSplus in exemplar MSK conditions [JIA, mucopolysaccharidoses (MPS), muscular dystrophy (MD), developmental coordination disorder (DCD) and healthy controls (HCs)]. The final pGALSplus was derived from analysis of phase 3 data and feedback from HCPs, families and expert consensus input from an international e-survey (*n* = 22) and virtual event (*n* = 13).

**Results:**

Feasibility was tested in 45 children (JIA, *n* = 10; MPS, *n* = 6; MD, *n* = 9; DCD, *n* = 10; HCs, *n* = 10). Overall the assessment was achievable in the target age range (2–10 years) and quick to complete [median 12 min (range 8–20)], with high acceptability from families. Expert feedback deemed pGALSplus to be very useful and of particular use to non-specialists in MSK paediatrics. The final pGALSplus comprises 26 clinical observations/skills with a colour-coding approach to aid decision-making and identification of more serious MSK presentations and additional resources to support its use in clinical practice.

**Conclusions:**

pGALSplus is a novel evidence- and consensus-based assessment building on pGALS, with high acceptability and feasibility. As community-based MSK assessment in children becomes more established, we propose that pGALSplus will facilitate and inform decision-making to promote access to specialist care.

Key messages

- pGALSplus aims to widen the scope of pGALS, supporting the recognition of serious MSK presentations.
- The pGALSplus assessment aims to facilitate onward referral pathways and may support identification of key MSK presentations.
- Our work proposes pGALSplus to be a novel evidence- and consensus-based assessment.

## Introduction

Musculoskeletal (MSK) symptoms in children are common (one in eight in the UK) [1], albeit with a spectrum of problems that are mostly benign and self-limiting, but there will be a minority with serious underlying conditions. Often children with MSK symptoms are initially assessed in primary or community care by healthcare professionals (HCPs) who may not be experts in MSK medicine or paediatrics. Pathways from primary or community care to specialist services are often complex, and delay in access to care is well reported in many MSK conditions [2–8]. Timely referral to the appropriate specialist can be critical for children affected by more serious MSK conditions, influencing early access to potential treatments and intervention and ultimately clinical outcomes.pGALS (paediatric gait, arms, legs and spine) [9, 10] is a simple, quick MSK assessment that is useful in clinical practice [11–17] and widely taught [18, 19]. While the pGALS assessment has been shown to detect joint and functional problems within the context of rheumatology, orthopaedics and neuromuscular medicine, alone it is unlikely to be specific enough to be diagnostic of any particular condition or be adequate to determine referral pathways to one specialist or another. Findings need to be interpreted in the clinical context and invariably this will entail further inquiry and physical or functional assessment [10, 13], currently beyond the scope of pGALS.

To address these challenges in clinical practice we developed the concept of ‘pGALSplus’ to widen the scope of the pGALS assessment, facilitate detection of serious MSK conditions and support decision-making about onward referral pathways [20]. This work included a review of the literature, which suggested that an MSK comprehensive assessment resource similar in type to pGALSplus does not currently exist.

Our concept of pGALSplus focused on development of an assessment that is simple and quick, for use with children 2–10 years of age (children within this age range may present with non-specific complaints and there is optimal opportunity for early intervention) and to be performed by physiotherapists and other HCPs integral to initial assessment and primarily working in the community and/or primary care. The aim of pGALSplus is to enable identification of children with potentially serious MSK conditions and aid HCPs in their decisions about subsequent referrals as necessary. In this article we discuss the iterative development of pGALSplus and evaluate its feasibility and acceptability in exemplar MSK presentations including inflammatory (JIA), neuromuscular [muscular dystrophy (MD)], neurodevelopmental [developmental coordination disorder (DCD)] and metabolic [mucopolysaccharidoses (MPS)] disorders. These exemplar conditions were chosen because they invariably present in young children with non-specific MSK complaints such as difficulties with balance, gross motor skills and functional skills such as standing from the floor, walking, climbing stairs and jumping. The initial clinical presentation is often challenging, crossing specialty boundaries, and there is evidence of delay in diagnosis for all [20]. As the main aims of this study were to assess the feasibility and acceptability of the pGALSplus assessment, psychometric properties including validity and reliability were beyond the scope of this research.

## Methods

A three-phase mixed methods approach was used.

### Phase 1: stakeholder engagement and scoping review

A preliminary stakeholder consultation event was held to engage HCPs within paediatrics and MSK medicine, generating ideas and addressing any possible barriers for change to their clinical practice. This event included representation from physiotherapy, occupational therapy, general practice, podiatry, neuromuscular and paediatric rheumatology specialists.

A literature review of the available evidence focused on key clinical assessments to inform diagnosis and subsequent referral [20].

### Phase 2: iterative development of pGALSplus

Interviews (*n* = 10) were conducted with expert HCPs (representatives from physiotherapy, occupational therapy, general practice, general paediatrics and specialist paediatrics) to identify key clinical assessments to inform diagnosis and progress. A focus group of HCPs working within physiotherapy and occupational therapy services (*n* = 8) enabled discussion to confirm additional tools to be included in pGALSplus. A summary report was discussed at a further expert workshop (*n* = 11 participants, including representatives from physiotherapy, paediatric rheumatology, neuromuscular specialists and general practice). A draft version of the pGALSplus assessment was thus created to be tested within phase 3.

### Phase 3: pGALSplus feasibility and acceptability pilot and evaluation

#### Pilot

An experienced paediatric physiotherapist (V.M.) used the pGALSplus assessment (including additional resources) in a sample of children with JIA (*n* = 10), MD (*n* = 9), MPS (*n* = 6), DCD (*n* = 10) and HCs (*n* = 10) at different ages (2–5 years, 6–10 years) to assess its feasibility and acceptability in clinical practice. The research team identified participants from their clinical practice with purposive sampling to include a range of ages (see Table 1). HCs were recruited through stakeholder contacts.

**Table 1.** Patient and HC demographics

Group	Age	Gender
JIA (*n* = 10)	Median 8 years 6.5 months (range 3 years 2 months–10 years 11 months)	Female: 4 (40%), male: 6 (60%)
MD (*n* = 9)	Median 6 years 8 months (range 5 years 4 months–9 years 3 months )	Female: 1 (11%), male: 8 (89%)
MPS (*n* = 6)	Median 7 years 9.5 months (range 5 years 5 months–10 years 4 months)	Female: 4 (67%), male: 2 (33%)
DCD (*n* = 10)	Median 8 years 11 months (range 7 years 6 months–10 years 7 months)	Female: 3 (30%), male: 7 (70%)
HCs (*n* = 10)	Median 4 years 8 months (range 2 years 1 month–9 years 9 months)	Female: 5 (50%), male: 5 (50%)

Evaluation focused on the feasibility and acceptability of pGALSplus, similar to methodology used to validate pGALS [9] (i.e. if children were able to complete the assessments, the time taken, acceptability to children and family; Likert-style ‘smiley’ face questionnaire assessing acceptability to the child and parent/guardian). A feedback questionnaire was also obtained from the physiotherapist undertaking the pGALSplus assessment, along with a reflective diary.

#### pGALSplus refinement

##### International e-survey

The proposed pGALSplus assessment was shared with international colleagues working in paediatric MSK medicine to consider the international healthcare context. The e-survey questions were piloted and tested before distribution with an e-mail reminder after 2 weeks. All survey responses were anonymized.

##### Stakeholder workshop

The pGALSplus assessment was further revised based on the findings from the pilot and international e-survey. The data and pGALSplus were presented at a final stakeholder event with representatives from the MSK and paediatric clinical communities (*n* = 13) (including representatives from physiotherapy, health visitor, podiatry, orthotics and neuromuscular medicine). Discussion focused on using pGALSplus within clinical settings and produced a list of recommendations for future practice as a result.

### Multimethod data analysis

The study received ethical approval from the Health Research Authority South Central – Hampshire A Research Ethics Committee (18/SC/0659 IRAS project ID: 246467). Written consent was obtained from all workshop, interview, focus group and pilot participants. Survey respondents consented to participation by submitting a completed response. All surveys, questionnaires, reflective diary responses and notes of participants’ discussions were anonymized. Interview and focus group data were audio-recorded with the participant’s consent and transcribed verbatim and transcripts formed the data subjected to formal analysis. The survey, questionnaire and reflective diary data were analysed using descriptive statistics and free-text comments using qualitative techniques. The workshop, focus groups and interview data were analysed qualitatively. All analysis of the qualitative data was conducted according to the standard procedures for qualitative analysis, including open and focused coding, constant comparison and deviant case analysis [21]. Data collection and analysis occurred concurrently, so that issues raised in earlier rounds of fieldwork could be explored in subsequent rounds. Reflexivity was maintained throughout the analysis and writing by recording, discussing and challenging established assumptions.

## Results

### Phase 1: stakeholder engagement and scoping review

Stakeholder engagement was positive, with constructive discussion around the need for a pGALSplus assessment. The scoping review identified specific assessment or screening tools (including a DCD questionnaire [22] and assessment of static balance) to add to pGALS. Thirty-five articles were deemed appropriate and are described elsewhere [20].

### Phase 2: iterative development of pGALSplus

Inclusion of assessment tools identified from the scoping review was discussed within the expert focus group, based on agreed upon criteria [tools excluded due to taking >15 min to complete, requiring specialist equipment or training, outside of the age range for the project (2–10 years), not transferable from a very specific client group, high cost, lack of sound evidence or poor expert opinion, lack of contextual relevance]. The practiced consensus was that pGALSplus should follow a structured approach, be quick to complete, user friendly and targeting a non-expert audience with a version for preschool children (ages 2–4 years) and another for school-age children (5–10 years) to consider different developmental norms. It was deemed important for pGALSplus to include ‘red flags’ that may indicate potential ‘life or limb limiting’ MSK conditions (e.g. septic arthritis, malignancies and slipped upper femoral epiphysis).

### Phase 3: pGALSplus feasibility and acceptability pilot and evaluation

On average, the assessment took a median of 12 min (range 8–20) to complete. Completion was marginally quicker when assessing the HCs and slower within the DCD patient group (Table 2).

**Table 2.** Average time taken to complete the pGALSplus assessment across the exemplar conditions and HCs

Characteristics	JIA	MPS	MD	DCD	HC	All
Participants, *n*	10	6	9	10	10	45
Median time (minutes)	10.5	12	12	17	10	12
Range (minutes)	9–13	10–15	10–15	10–20	8–15	8–15

Most assessments were judged to be ‘easy’ or ‘very easy’ for the physiotherapist to undertake (96%: easy 38%, very easy 58%) and for the child to undertake (84%: easy 53%, very easy 31%). Most children (84%) reported no discomfort, and for those who did, this was deemed by the physiotherapist (expert opinion) to be due to the nature of their condition (i.e. a flare up of JIA, muscular pain and fatigue associated with MD or tight muscle groups (hamstrings) in DCD) rather than a component of pGALSplus itself. For the small number of children for whom the assessment was not deemed to be ‘easy’, this was considered likely due to young age or their level of understanding. For some children, elements of the assessment were ‘difficult’ or unable to be completed (see Table 3); for children with JIA, skills such as catching a ball or balance when hopping and standing on one leg were difficult if they had joint disease in those areas. Some children with MPS had contractures of their elbows, wrists and fingers, which made skills such as removing their T-shirt, raising their arms above their head and catching a ball more challenging. Boys with MD struggled with movements against gravity, such as raising their head from the floor and standing up from the floor. Within the DCD group, some children struggled to follow instructions or complete the task requested and found ball skills and sequencing (jumping sequence) more challenging, most likely due to motor planning difficulties. Reflective diary notes recorded by the physiotherapist confirmed these challenges and were used to aid the iterative development of pGALSplus and to inform a ‘basic instructions’ resource. Difficulties such as side flexion of the cervical spine (ear to shoulder) and lumbar spine flexion (bending forward to touch toes) identified in the HC group were deemed likely due to a lack of understanding of the instructions in the younger age group and led to iterative development of the preschool pGALSplus (2–4 years).

**Table 3.** Feasibility of pGALSplus clinical observations and skills across the disease groups (and citing those who were reported to be ‘difficult’ across the groups)

pGALSplus clinical observation/skills	Condition group and the children observed to have an abnormality or difficulty with each clinical observation/skill, *n* (%)				
	JIA (*n* = 10)	MPS (*n* = 6)	MD (*n* = 9)	DCD (*n* = 10)	HCs (*n* = 10)
Observation: take T-shirt off	1 (10)	4 (67)	3 (33)	2 (20)	0
Observation: standing posture (swelling, alignment, rash, deformity)	0	5 (83)	2 (22)	0	0
Walk on heels then tiptoes	4 (40)	4 (67)	8 (89)	4 (40)	1 (10)
Hold hands out straight	0	6 (100)	1 (11)	0	0
Make a fist	0	0	0	0	0
Pinch index finger and thumb	1 (10)	2 (33)	0	0	0
Squeeze MCP joints	0	0	0	0	0
Hands: palm to palm/back to back	0	5 (83)	1 (11)	0	0
Reach arms up, touch the sky, head back	0	5 (83)	2 (22)	0	0
Hands behind neck	0	5 (83)	1 (11)	0	0
Touch shoulder to ear	0	0	0	0	1 (10)
Three fingers (own) in mouth	0	1 (17)	0	0	1 (10)
Feel for effusion in knees	4 (40)	1 (17)	0	0	0
Active movement of knees	0	1 (17)	3 (33)	0	0
Leg length discrepancy (≥1 cm)	2 (20)	0	0	0	0
Passive movement of hips	2 (20)	1 (17)	0	0	0
Lower limb reflexes	0	0	0	0	0
Bend forward and touch toes	1 (10)	3 (50)	3 (33)	0	1 (10)
Head raise in supine position/pull to sit		6 (100)	6 (67)	0	0
Rise from the floor	1 (10)	4 (67)	8	1 (10)	0
Functional squat and rise	0	2 (33)	6 (67)	0	0
Stand on one leg (eyes open)	4 (40)	5 (83)	8 (89)	3 (30)	1 (10)
Stand on one leg (eyes closed)	4 (40)	3 (50)	4 (44)	9 (90)	0
Hop	3 (30)	5 (83)	8 (89)	5 (50)	0
Jump	1 (10)	3 (50)	8 (89)	5 (50)	
Ball skills	1 (10)	3 (50)	5 (56)	7 (7)	2 (20)
Total reporting abnormality/difficult	8 (80)	6 (100)	9 (100)	10 (100)	3 (30)

All families reported the time taken to complete the assessment was ‘about right’ and most (69%) found the assessment ‘very comfortable’. Of the 24% of children who reported some ‘discomfort’ or ‘pain’ (Table 4), as discussed above, this was deemed to be due to the nature of their condition rather than the component of pGALSplus itself. Additional comments were invariably positive, with the examination noted to be quick, thorough, appropriate, engaging, enjoyable, play focused and child friendly. This was also reinforced by the reflective diary entries of the physiotherapist.

**Table 4.** Parent/guardian and/or child reported level of comfort during pGALSplus assessment

Level of comfort, *n* (%)	JIA	MPS	MD	DCD	HC	All
Very painful	–	–	–	–	–	–
A little pain	–	–	1 (11)	1 (10)	–	2 (4)
A little discomfort but no pain	3 (30)	1 (17)	2 (22)	3 (30)	–	9 (20)
It was ok	–	–	–	1 (10)	2 (20)	3 (7)
Very comfortable, no pain	7 (70)	5 (83)	6 (67)	5 (50)	8 (80)	31 (69)
Total, *n*	10	6	9	10	10	45

####  

##### International e-survey and final stakeholder event

Purposive sampling recruited 22 international colleagues (identified from previous research collaborations [23–26]) working in paediatric rheumatology (*n* = 15), adult rheumatology (*n* = 2), physiotherapy (*n* = 4) and podiatry (*n* = 1), with their feedback through e-surveys [using SurveyMonkey (https://www.surveymonkey.com/)]. Overall, expert feedback (international e-survey, *n* = 22; stakeholder event, *n* = 13) was positive and deemed pGALSplus to be relevant to the practice of non-MSK specialists, physiotherapists with a predominant adult focus, those new to the paediatric specialty and other professionals, including orthotists and health visitors. Recommendations included colour-coding to streamline the flow of the assessment and format changes to the proforma for ease of use. It was deemed important to emphasize that the assessment does not require any specialist equipment (e.g. a tennis ball could easily be substituted with any small light object).

## Discussion

We believe that pGALSplus is a novel assessment tool that could support HCPs in the recognition of children with more serious MSK presentations, aiding decision-making and informing appropriate referral pathways. The need for pGALSplus arose from the challenges of clinical practice [20] and evidence of delay in the diagnosis of serious MSK conditions. Through a literature review and expert opinion, we identified additional components to be added to pGALS to produce a comprehensive MSK assessment in the context of exemplar conditions. While not diagnostic, pGALSplus enables HCPs who may not be experienced in MSK paediatrics to recognize those children with presentations that warrant further investigation, facilitating early identification and intervention. Stakeholder discussion allowed refinement and agreement on how pGALSplus would be used in practice and identified key components for inclusion. The final consensus agreed pGALSplus should include 26 clinical observations and skills, with a colour-coding approach to facilitate identification of exemplar MSK conditions. Signposting to additional resources and a ‘red flag’ list allows professionals to identify ‘life or limb’ limiting conditions. Two versions of pGALSplus were developed—one for preschool children (ages 2–4 years) and another for school-age children (5–10 years)—to consider different developmental norms (see Supplementary Data S1 and S2, available at *Rheumatology Advances in Practice* online). Table 5 provides a summary of how to complete pGALSplus in practice.

**Table 5.** Recommendations on how to complete pGALSplus

Assessment instructions/tips
Introduction
Introduce self. Explain pGALSplus assessment to child and parent/caregiver. Explain that the assessment should only take 10–15 min. Obtain consent from parent/caregiver. Check child is wearing appropriate clothing—shorts (and T-shirt or vest if preferred), shoes and socks removed.
Equipment
The pGALSplus assessment is designed to use minimal equipment that is commonly found in any therapy/rehabilitation department or paediatric clinic setting. The assessment can be used in different clinical settings. It will be useful to have: Examination plinth/couch or bed Tennis ball (or a small ball) and football (or suitable equivalent such as a soft massage ball) Reflex/tendon hammer Mat for the floor (if possible, but can be completed without).
Generic skills
Use age-appropriate language (not jargon) when giving instructions to patients. Use ‘copy me’ where appropriate. Assessment best performed in a systematic and fluent manner (head to toe approach). pGALSplus school-age and preschool assessments are very similar but include some alternative age-related skills.
Completing the pGALSplus assessment
Complete the pGALSplus assessment using the combined assessment and recording proforma. If there are no concerns, this should be recorded as a **✓** in the appropriate boxes. Record any concerns as an X in the appropriate boxes, with comments and observations. Remember to score ALL coloured boxes that apply (this may be up to six colours for some observations). Once you have completed the assessment, add your scores to the white boxes in the tables on pages 5 and 6; you can also summarize your findings on page 4. Begin with the colour with the highest score and use the coloured tables on pages 5 and 6 to guide you; i.e. mostly yellows, use the yellow table. If scores are high for more than one colour, consider using all of the resources that may guide you further. Make use of additional signposted resources and consider onward referral if appropriate. Make use of the case scenarios available that illustrate examples of the scoring process.
Ask screening questions
Ask the screening questions at the start of the assessment. These can be asked to both the child and the parent/caregiver. Record any comments in the boxes.
General observations
Ask the patient to remove his/her T-shirt. Observe how they do this and note any difficulty due to lack of range of movement, weakness in the upper limbs or difficulty with motor planning. The child can then put his/her T-shirt back on if this is more comfortable for them. Within the pre-school assessment, children can be assisted with this skill. Observe the patient in standing from the front, back and side; comment on posture, asymmetry, spine alignment and foot position.
Gait
Observe the patient walking. Ask the patient to walk on tiptoe and then on heels. Look for restriction in the range of movement at the ankle, weakness around the ankle and foot and difficulty understanding instructions. For preschool children, look at tiptoe in standing.
Arms
Ask the patient to put hands out in front, palms down, fingers outstretched. Ask the patient to turn hands over and make a fist. Ask the patient to pinch his/her index finger and thumb together. Squeeze the metacarpophalangeal joints (check for discomfort). Ask the patient to put hands and wrists together—palm to palm—wrists at full extension, fingers straight and pointing upwards. Ask the patient to put hands back to back, wrists in full flexion, fingers straight and pointing downward. Ask the patient to reach up as far as he/she can with arms straight. Ask the patient to put hands behind head, pushing elbows back.
Legs
Ask the patient to lie in a supine position on the plinth. Check general appearance of legs—muscle bulk, swelling, asymmetry. Feel for effusion at the knee (patella tap, cross fluctuation). Ask the patient to fully extend and then flex the knee, then repeat with the other knee. Check passive range of movement at the knee and feel for crepitus. Check for any leg length discrepancy and measure if appropriate. With hip and knee flexed to 90 degrees, check the internal and external rotation of the hip. Check lower limb reflexes—patella, ankle and Babinski. Note any brisk or absent reflexes, upgoing plantar reflex.
Spine
Comment on alignment of the spine—scoliosis, lordosis, kyphosis. Ask the patient to look at the ceiling (neck extension). Ask the patient to place each ear to his/her shoulder (or turn head to left and right for preschool). Ask the patient to bend forwards and touch their toes (this can be done in long sitting for preschool). Ask the patient to open his/her mouth wide and place three fingers vertically inside their mouth (or as wide as they can for preschool).
Plus
Ask the patient to lie on his/her back on the mat/floor. Ask him/her to cross arms over the chest and lift his/her head up. Look for midline position. In preschool assessment, hold the patient’s upper limbs and pull them into a sitting position; check for any head lag. Ask the patient to stand up from the floor as quickly as he/she can. Look for any difficulty standing, Gowers’ manoeuvre in an older child and restriction in the range of movement. For preschool children this should not be timed. Ask the patient to pick something up from the floor by bending his/her knees and squatting down. Ask the patient to stand on one leg with arms out to the side; repeat on the other leg. For older children, try this with eyes closed. Very young children could be asked to kick a ball. Ask the patient to hop on one leg (up to 10 times) then repeat on the other leg. Ask the patient to jump forwards three times with both feet together. For older children, add in a sequence of jumps (forward, back and to the side). Ask the patient to throw and catch a small ball (tennis ball) with the assessor. Younger school-age children should do this two hands together, older children should use their dominant hand. For preschool, use a larger ball (football) and ask the patient to throw it.

Our work has demonstrated the pGALSplus assessment is quick, easy and achievable in the target groups with high levels of acceptability from families. We included children with grades of condition severity across the exemplar groups and, as expected, those children who were more significantly affected did find elements of the assessment difficult. For example, children at the upper end of the age group with MD had more severe disease and found skills such as rising from the floor more challenging. Likewise, older children in the MPS group with more advanced disease struggled with skills such as taking their T-shirt off, due to contractures of the upper limbs. Children with JIA involving upper limb joints found catching a ball to be difficult. Final expert feedback deemed pGALSplus to be a very useful addition to pGALS, allowing non-MSK specialists and those new to the paediatric specialty to recognize children who may have a more serious underlying disorder and to plan appropriate specialist referrals.

### Limitations

We recognize that our chosen condition groups are exemplars, and although not generalizable to all MSK conditions, we included a range of MSK pathologies: inflammatory (JIA), neuromuscular (MD), metabolic (MPS) and developmental (DCD), as well as an HC cohort. All of the exemplar conditions may present in younger children, with non-specific complaints (such as difficulties with balance and gross motor skills and functional skills such as standing from the floor, walking, climbing stairs and jumping). Initial presentations may cross clear specialty boundaries, and there is evidence of delay in diagnosis for all. Conditions such as MPS are rare and therefore a small sample size is inevitable. We also recognize that for the purpose of the pilot, the assessment tool was administered by a physiotherapist with experience working with children with MSK diseases, and therefore this may not completely reflect how the assessment would be performed by HCPs with more limited proficiency. Feedback suggested that use of the pGALSplus tool and proformas was straightforward and we do not envisage there being a requirement for health professionals to complete further training. However, we have developed resources to aid implementation. These include a graphic animation on how to complete the assessment in practice, a ‘top tips’ guide and a completed assessment sample. These resources are free and openly available to all (www.pmmonline.org/doctor/clinical-assessment/examination/pgalsplus).

Our work has developed pGALSplus as a novel evidence- and consensus-based assessment with high acceptability and feasibility that may support recognition of key MSK conditions in children. Further work is needed to assess both the reliability and validity of the assessment, identify diagnostic accuracy and inform decision-making about onward referral to specialists. We envisage pGALSplus being of particular use in the primary and community care settings by allied health professionals who may not be experts in paediatric MSK practice, allowing them to identify when it may be appropriate for a child to be referred onwards and, based on clinical assessment findings, to which specialist service. There may also be the potential for pGALSplus to be used in conjunction with artificial intelligence to guide decision-making/diagnosis and referral.

The challenge in clinical practice is getting the right child, to the right place at the right time. Safe, effective triage of children with MSK problems by paediatric physiotherapists has been demonstrated in primary care [26]. In the UK, NHS England has produced a framework to reduce community-based MSK waiting times while delivering the best outcomes for patients [27]. This involves the utilization of MSK physiotherapists in a first-contact practitioner role working with adults, and as this is likely to expand into paediatrics, we see an increasing role for the pGALSplus tool in clinical practice.

## Supplementary Material

rkae089_Supplementary_Data

## Data Availability

The data underlying this article are available in the article and in its online supplementary material.
