# Supplementary material for: The development of pGALSplus: evaluating feasibility and acceptability of an assessment to facilitate the identification and triage of children with musculoskeletal presentations
Source: Rheumatol Adv Pract. 2024 Aug 1;8(3):rkae089. doi: 10.1093/rap/rkae089 (PMC11319642; doi:10.1093/rap/rkae089)
Supplement: rkae089_Supplementary_Data [file rkae089_supplementary_data.zip › 24-046 Supplementary Data S1.pdf]

pGALSplus

Version 1 – School age (5 to 10 years)

Name……………………………. D.O.B…………………… NHS Number………………… Red flag checklist completed Y/N

Parent's concerns **Y/N**	Child/Young Person's concerns **Y/N**
………………………………………………………………………………	………………………………………………………………………………

Answer the following screening questions and then complete the assessment. **Record any concerns as an X in the appropriate boxes, or a ✓ if no concerns**, with comments/observations as appropriate. **Score <u>ALL</u> coloured boxes that apply, then add the colour totals to the summary boxes at the end of the assessment**

	✓	Difficulty/Restriction due to reduced ROM, contracture, swelling or pain	Difficulty/abnormality due to weakness/asymmetry/ poor quality of movement	Difficulty due to understanding/ clumsiness/ motor planning	Difficulty due to other observations e.g. hypermobility
Observation: Take T shirt off **Look for any difficulties** 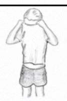		*Example* X ↓ ROM at shoulders			
Observation: Standing posture **Swelling, alignment, rash, deformity** 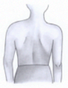					

The following questions can be answered by the child or young person, or by a parent or guardian on behalf of the child.

	No concerns✓	Comments
Do you (or does your child) have any pain or stiffness in their joints, muscles or back, that is impacting on your (their) daily life? If yes, does the pain change over the course of the day?		
Do you (or does your child) have any difficulty getting yourself (him/herself) dressed without any help?		
Do you (or does your child) have any difficulty walking from one place to another (for example walking to school)?		
Do you have (or have you ever had) any concerns about your child's development (either gaining new skills or losing established skills)?		
When you think back, are there any skills that you have tried to learn (or teach your child to do) that have taken longer than you think it should have (for example tying shoe laces, riding a bike, playing a ball game)?		
With regards to everyday activities such as running and jumping, can you (or can your child) keep up with other children of a similar age?		
Would you ever describe yourself (or your child) as being 'accident prone' or more likely to have trips and falls?		

pGALSplus

Version 1 – School age (5 to 10 years)

	✓	Difficulty/Restriction due to reduced ROM, contracture, swelling or pain	Difficulty due to weakness/asymmetry/ poor quality of movement	Difficulty due to understanding/ clumsiness/ motor planning	Difficulty due to other observations e.g. hypermobility
Observation:<br>Take T shirt off<br>**Look for any difficulties** 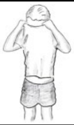					
Observation:<br>Standing posture<br>**Swelling, alignment, rash, deformity** 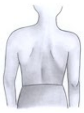					
Walk, walk on heels then tiptoes<br>**Look at ability and foot posture, mirroring of ULs** 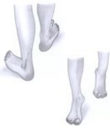					
Hold hands out straight<br>**Elbow, wrist, finger extension** 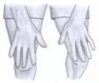					
Make a fist<br>**Supination, flexion of fingers** 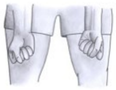					
Pinch index finger and thumb<br>**Look at finger joints, functional grip** 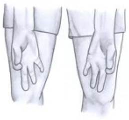					
Squeeze MCP joints<br>**Assess for tenderness** 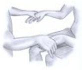					
Hands –<br>Palm to palm/back to back<br>**Wrist flexion and extension** 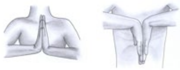					
Reach arms up, touch the sky, head back<br>**Elbow, wrist, neck extension** 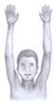					
Hands behind neck<br>**Shoulder abduction, external rotation** 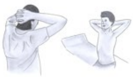					
Touch ear to shoulder<br>**Cervical spine side flexion** 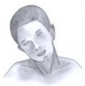					
Three fingers (own) in mouth<br>**Temporomandibular joints** 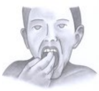					
Feel for effusion in knees<br>**Patella tap, cross fluctuation** 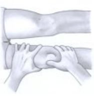					

pGALSplus

Version 1 – School age (5 to 10 years)

	✓	Difficulty/Restriction due to reduced ROM, contracture, swelling or pain	Difficulty/abnormality due to weakness/asymmetry/ poor quality of movement	Difficulty due to understanding/ clumsiness/ motor planning	Difficulty due to other observations e.g .hypermobility
Active movement of knees<br>**Knee flexion/extension** 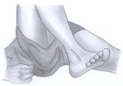					
Leg length discrepancy (1cm or more)<br>**Eyeball or can measure** 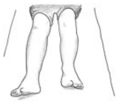					
Passive movement of hips<br>**Hip flexion/internal and external rotation** 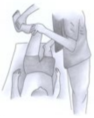					
Lower limb reflexes<br>**Patella, ankle, Babinski** 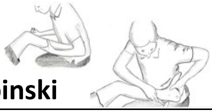					
Bend forwards and touch toes<br>**Forward flexion of spine** 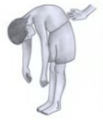					
Head raise in supine<br>**Chin towards chest in midline** 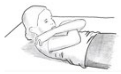					
Rise from the floor (from supine) in less than 3 seconds<br>**Look for Gowers', quality of movement, use of furniture** 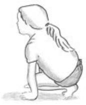					
Functional squat to floor and rise (to retrieve toy) 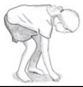					
Standing on one leg right and left **eyes open**<br>***age 5-6*** up to 10 seconds<br>***age 7-10*** > than 10 seconds 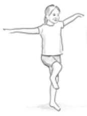					
Stand on one leg R and L **eyes closed (aged 7-10 only)**					
Hopping<br>***age 5-6*** up to 5 hops<br>***age 7-10*** > 5 hops 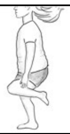					
Jump 2 feet together<br>**Age 5-6** 3 times<br>***age 7-10*** (3 forward, 2 back, 1 left) 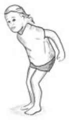					
Ball skills -throw and catch small ball<br>***Age 5-6*** 2 times (both hands)<br>***Age 7-10*** 5 times (dominant hand) 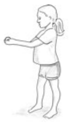					

Version 1 – School age (5 to 10 years)

pGALSplus

Screening questions	Comments
Do you (or does your child) have any pain or stiffness in their joints, muscles or back, that is impacting on your (their) daily life? If yes, does the pain change over the course of the day?	
Do you (or does your child) have any difficulty getting yourself (him/herself) dressed without any help?	
Do you (or does your child) have any difficulty walking from one place to another (for example walking to school)?	
Do you have (or have you ever had) any concerns about your child's development (either gaining new skills or losing established skills)?	
When you think back, are there any skills that you have tried to learn (or teach your child to do) that have taken longer than you think it should have (for example tying shoe laces, riding a bike, playing a ball game)?	
With regards to everyday activities such as running and jumping, can you (or can your child) keep up with other children of a similar age?	
Would you ever describe yourself (or your child) as being 'accident prone' or more likely to have trips and falls?	

+

Observations and Examination			
	Observations/Appearance (✓ if no concerns), X if concerns	Clinical findings	Comments
Posture			
Gait			
Arms			
Legs			
Spine			
Plus (Function, skill, movement)			

pGALSplus

Mostly yellows – could indicate an inflammatory disorder such as Juvenile Idiopathic Arthritis
Further examination of swelling or signs of inflammation in joints

Score

+

Additional Tools
Recognition from visual appendices (swollen joints, fixed deformities, compare to MPS)
paediatric musculoskeletal matters pREMS assessment
Consider hypermobility BSR Guidance/RCPCH Position Statement

=

Refer to Paediatric Rheumatology Service

Mostly oranges – could indicate a Lysosomal storage disorders such as Mucopolysaccharidoses
Stiffness or contracture of upper limb joints, in the absence of swelling can be a sign of a lysosomal storage disorder, such as the mucopolysaccharidoses

Score

+

Additional Tools
Recognition from visual appendices (restriction of UL joints in the absence of swelling, gibbus deformity, compare to JIA
Resources from MPS Society MPS Society Resources
paediatric musculoskeletal matters pREMS assessment
Consider hypermobility BSR Guidance/RCPCH Position Statement

=

Refer to Specialist Genetic Centre

Mostly blues – could indicate a neuromuscular disorder such as Duchenne muscular dystrophy
Assess for functional muscle weakness and delay/regression in motor milestones

Score

+

Additional Tools
Recognition from visual appendices (calf hypertrophy, Gowers' sign, scapular winging)
Think MUSCLE<br>Fox et al., (2020) BMJ;368:l7012
Treat -NMD Neuromuscular network treat-nmd.org

=

Refer to Specialist Genetic Centre

Version 1 – School age (5 to 10 years)

**Mostly purples – could indicate an underlying neurological disorder or an orthopaedic condition**<br>Further assessment of asymmetry of limbs and spine, muscle tone, muscle bulk, range of movement.	Score

+

Additional Tools
Further assessment of muscle tone
paediatric musculoskeletal matters pREMS assessment
Hip rotational profiles Staheli et al., (1985) The Journal of Bone and Joint Surgery 67(1) 39 (image available at Researchgate.net)

=

Refer to General/Community Paediatrician or Orthopaedic Surgeon

**Mostly greens – issues with motor planning and gross/fine motor skills**<br>This may warrant further assessment by Paediatric Physiotherapy and/or Occupational Therapy, or a general paediatrician	Score

+

Additional Tools
Screening activities and gross motor chart Missiuna et al., (2006) CMAJ 175 (5) 471
CanChild resources for professionals CanChild.ca
DCDQ-07 Questionnaire Wilson and Crawford, (2012) Physical and Occupational Therapy in Pediatrics
Consider hypermobility BSR Guidance/RCPCH Position Statement

=

Refer to local Physiotherapy/Occupational Therapy Service or Paediatrician

**Mostly pinks – issues with pain, balance and motor skills may indicate hypermobility**<br>If no evidence of serious pathology, offer advice, education and reassurance	Score

+

Additional Tools
paediatric musculoskeletal matters pREMS assessment
Consider hypermobility BSR Guidance/RCPCH Position Statement

=

Reassurance, education and advice, onward referral if necessary
